# The New Medical Benefit Regulations

**Published:** 1915-12-04

**Authors:** 


### NATIONAL INSURANCE ACT DEVELOPMENTS

### THE NEW MEDICAL BENEFIT REGULATIONS.

It will, doubtless, be remembered that the Regulations governing the administration of medical benefit under the National Insurance Acts were to have been revised as from the commencement of the present year, but owing to the war it was found impossible to make the alterations at that time, with the exception that a uniform system of certification was then adopted. In spite of the fact that the war is still being waged, it has now been decided by the Insurance Commissioners that certain important alterations are to be made in the Regulations as from January 1 next. This decision is made known by the issue of draft Regulations, dated October 27, which will in due course become substantive. With these draft Regulations there has also been issued a Memorandum to Insurance Committees (Memo. 217/I.C.) calling attention to the principal alterations that are contemplated, and giving an account of the reasons for making the revision. These two documents are of considerable interest, and are probably the most important that have recently been issued by the Insurance Commissioners.

### The Insufficiency of the Drug Funds.

It is made clear in the Memorandum that the necessity for revision of the Medical Benefit Regulations has arisen mainly owing to the situation created by the recently published Report of the Departmental Committee upon the drug tariff. That Committee was formed in order to investigate the question of the alteration of the method of paying the chemists for drugs supplied to insured persons, in view of the serious advance in the price of most drugs that has occurred owing to the war. They also had to consider, incidentally, the question of excessive prescribing.

In their Report the Committee recognised that the only equitable solution of the difficulties that had arisen was the introduction of a tariff based upon commercial prices of the drugs supplied, and they recommended the adoption of such a plan. This recommendation led necessarily to the condition that, as the prices were to be commercial, each chemist's account would have to be paid in full, and there could no longer be any possibility of the proportionate reduction of accounts, as under the present system, in the event of the Drug Fund for the particular Insurance Committee area being insufficient to meet the accounts in full.

It will, of course, be remembered that under the existing Regulations there is one fund out of which both the doctors' and the chemists' accounts are satisfied. The fund is represented by 9s. for each insured person per annum, and the division as between the doctors and the chemists is that the former are entitled to a minimum sum of 7s. for each patient, and the latter in the aggregate to a minimum sum of 1s. 6d. The difference between the 9s. and the allocated minimum of 8s. 6d. has generally been termed the "floating sixpence." If the chemists' accounts did not in the aggregate, for any particular Insurance Committee area, exceed the sum of 1s. 6d. per insured person within that area, the "floating sixpence" was made available for distribution among the doctors; otherwise such part of the "floating sixpence" as was necessary had to be devoted towards the payment of the chemists' accounts. It will at once be seen that the chemists' maximum claim on the fund was 2s. per insured person, and if the chemists' accounts were to be paid in full on a commercial basis it would be necessary either to reduce the minimum sum of 7s. due to the doctors or else to provide some means for augmenting the Medical Benefit Fund, in the event of the chemists' accounts exceeding 2s. per person.

### The Commissioners' Dilemma.

The Insurance Commissioners, in adopting the commercial basis for the chemists' accounts, were of opinion that a substantial reduction would be made in the pricing of certain drugs and that, apart from sheer extravagance in prescribing, there was really very little risk of the doctor's 7s. being encroached upon.

When, however, the Commissioners approached representatives of the medical profession they found them unwilling to run the risk of any reduction below the existing agreed minimum. By this rejection of the Commissioners' proposals it was also implied that the doctors did not wish to gain any advantage which might accrue to the Drug Fund by reason of the adoption of the commercial tariff.

The Insurance Commissioners were thus placed in a position of considerable gravity. Having admitted the principle that the chemists should be paid in full on a commercial basis they could not resort to the expedient of postponing the revision of the Regulations, more especially as there was a serious risk that the chemists would not renew their agreements.

### A Solution of the Difficulty.

A solution of the difficulty was ultimately found by obtaining from the Treasury guarantees whereby it is hoped that both the doctors and the chemists will be satisfied. The arrangements made are briefly that (1) the doctors will be guaranteed their minimum of 7s.; and the present arrangement will continue for the division of the "floating sixpence" on the basis of the existing drug tariff (not the commercial tariff); (2) by pooling the balances of the Drug Fund remaining in each area into one central fund the chemists will be guaranteed the payment in full of their accounts on the basis of the new commercial tariff, and the Treasury take the risk of having to make good any deficiency that may arise.

It will at once be noticed that the new scheme will require that every prescription will have to be priced on two scales. This is, so far as we can see, the only disadvantage of the plan. It means extra clerical labour on the part of the chemists and also for the Insurance Committees, but this extra work will be compensated by the satisfaction which will be given to the chief contracting parties. If no claim is made upon the Treasury for making good deficiencies it will be clear that the commercial tariff would have benefited the doctors, while, on the other hand, if the Treasury has to meet any substantial demand under the terms of its guarantee the doctors will be able to congratulate themselves on their decision.

### Prescriptions and Certificates.

The new Regulations also provide for some minor alterations in procedure, which, though relatively of much less importance than the change of basis already indicated, are calculated to facilitate the working of the Acts. Thus, for instance, it is provided that panel doctors may no longer give prescriptions which necessitate reference on the part of a chemist to a previous order. In other words "repeat prescriptions" are to terminate. Prescriptions of this class have long been resented by the panel chemists whose views in this respect the new Regulations meet. It is provided that the medical certificates that have to be furnished for the purpose of enabling insured persons to claim benefit from their approved societies "shall be written in ink or in some other indelible substance, and the practitioner's signature shall be written with his own hand." The intention is to preclude the possibility of certificates being tampered with or issued by unauthorised persons.

